# Global Tractography with Embedded Anatomical Priors for Quantitative Connectivity Analysis

**DOI:** 10.3389/fneur.2014.00232

**Published:** 2014-11-17

**Authors:** Alia Lemkaddem, Didrik Skiöldebrand, Alessandro Dal Palú, Jean-Philippe Thiran, Alessandro Daducci

**Affiliations:** ^1^Signal Processing Laboratory (LTS5), École Polytechnique Fédérale de Lausanne, Lausanne, Switzerland; ^2^Department of Mathematics and Computer Science, University of Parma, Parma, Italy; ^3^Department of Radiology, University Hospital Center and University of Lausanne, Lausanne, Switzerland; ^4^Center for Biomedical Imaging, Signal Processing Core, Lausanne, Switzerland

**Keywords:** diffusion MRI, global tractography, spline, brain connectivity, MCMC, anatomical priors

## Abstract

Tractography algorithms provide us with the ability to non-invasively reconstruct fiber pathways in the white matter (WM) by exploiting the directional information described with diffusion magnetic resonance. These methods could be divided into two major classes, local and global. Local methods reconstruct each fiber tract iteratively by considering only directional information at the voxel level and its neighborhood. Global methods, on the other hand, reconstruct all the fiber tracts of the whole brain simultaneously by solving a global energy minimization problem. The latter have shown improvements compared to previous techniques but these algorithms still suffer from an important shortcoming that is crucial in the context of brain connectivity analyses. As no anatomical priors are usually considered during the reconstruction process, the recovered fiber tracts are not guaranteed to connect cortical regions and, as a matter of fact, most of them stop prematurely in the WM; this violates important properties of neural connections, which are known to originate in the gray matter (GM) and develop in the WM. Hence, this shortcoming poses serious limitations for the use of these techniques for the assessment of the structural connectivity between brain regions and, *de facto*, it can potentially bias any subsequent analysis. Moreover, the estimated tracts are not quantitative, every fiber contributes with the same weight toward the predicted diffusion signal. In this work, we propose a novel approach for global tractography that is specifically designed for connectivity analysis applications which: (i) explicitly enforces anatomical priors of the tracts in the optimization and (ii) considers the effective contribution of each of them, i.e., volume, to the acquired diffusion magnetic resonance imaging (MRI) image. We evaluated our approach on both a realistic diffusion MRI phantom and *in vivo* data, and also compared its performance to existing tractography algorithms.

## 1 Introduction

Since the introduction of diffusion Magnetic Resonance Imaging (MRI) (1), the technique has been exploited in clinical and research studies as it allows to assess the micro-structural integrity of the neuronal tissue in the brain. *Tractography* is a general term for a class of methods to reconstruct the trajectories of the fibers in the WM based on local information about the neuronal tissue estimated from diffusion MRI (dMRI) data. These algorithms offer a unique possibility to gain insight into the structure of the human brain non-invasively and *in vivo*. The information gained in this manner is not only of high value for visualization of the brain connectivity and segmentation of the brain into different functional areas, but also has the potential to provide essential information that can be exploited, e.g., for neurological planning or for better understanding major diseases such as multiple sclerosis, epilepsy, schizophrenia, brain plasticity after strokes, etc.

Most of the tractography algorithms proposed to date are based on *local* approaches, meaning that they consider only local diffusion information as a streamline is propagated throughout the WM. These algorithms, can be either deterministic (2, 3) or probabilistic (4, 5). The simplest approach reconstructs the neuronal pathways by following the local, voxelwise defined diffusion direction in small successive steps. Despite being very fast, these approaches suffer from the fact that integration errors accumulate along the path and can lead to great deviations from the true underlying fiber trajectory. Probabilistic methods extend these methods by estimating a distribution of possible pathways; a streamline is continued by drawing samples from this distribution (4). Often, the number of probabilistic streamlines generated, when compared to an equivalent experiment using deterministic streamlines, needs to be much larger. Probabilistic methods come with a significantly higher computation time together with an increased chance of generating false positive pathways and, especially, do not solve the intrinsic limitations of the local schemes. Therefore, to overcome the local nature of previous approaches, front-evolution methods have been introduced (6, 7). In these methods, the local diffusivity can be interpreted as local speed. Paths with higher diffusivity are traversed with higher speeds than paths of low diffusivity. Thus, the global optimal connection between two regions can be thought of as the path with the minimal arrival time. These techniques bring us closer to a *global* approach that are computationally efficient. However, for any pair of regions in the brain, there exists a geodesic between two regions. Meaning that all the regions in the brain can be connected to each other, which is not anatomically possible. Again as in the case of the probabilistic approach a high number of false positive fibers are introduced. Recently, *global* energy minimization techniques (8–10) fall within the category of global tractography. The aim of these methods is to reconstruct the complete tractogram by integrating all the diffusion information of the brain. As a result, these global algorithms show improvements compared to previous methods (11), but the price to pay is the increased computational burden.

Today, most existing algorithms suffer from two major drawbacks that limit their effectiveness with respect to connectivity analyses: firstly, most fibers stop prematurely in the WM, which violates a very important anatomically property of neuronal connections. This has already been addressed in recent work for *local* approaches (12, 13). However, in the context of global tractography this problem has not been taken into consideration. Furthermore, a comparison study (14) of a large collection of tractography algorithms and local reconstruction methods based on the FiberCup dataset (15), shines a light on this ambiguity. The authors show that indeed between 58 and 97% of the reconstructed fibers do not reach the GM. This issue has been also highlighted in human brain data by (16), who showed that one-third of the fibers do not connect to the GM, meaning that these connections stop prematurely in the WM and thus, are of no help in structural connectivity analyses. Secondly, the reconstructed trajectories are not quantitative (17, 18). The counts for number of streamlines connecting two regions in the brain demands some normalization that are hard to justify and averaging along some scalar values (e.g., FA) is only an indirect measure of the underlying neuronal-structure. Recent studies have been devoted to deal with this issue (19–21), but the proposed implementations are very burdensome to be used in practice. Ref. (22) has recently proposed a general and very efficient framework to combine tractography and tissue micro-structure estimation using a convex formulation. Thus, leading to a more quantitative and biologically oriented assessment of brain connectivity. Nevertheless, all existing approaches assume an input set of tracts whose positions are fixed and cannot be adapted. As a consequence, all these formulations are sensitive to the choice of the algorithm used to estimate the candidates fibers.

In this work, we propose a method that re-addresses the importance of anatomical priors in global tractography by exploring the different states of the fiber model to find the combination which best explains the data. This is achieved by first expressing the fiber pathways as splines, which are described by their control points, where the two extreme points are placed in the GM and the rest are constrained in the WM. Also, the splines are smooth by nature and therefore there is no need for additional constraints to force the shape of a plausible fiber. Furthermore, since the fibers are changed during the optimization process there is no big dependency of the initial solution (candidate fibers). Lastly, every fiber is associated with a weight, which brings us closer to a quantitative tractogram.

## 2 Methods

### 2.1 Proposed approach

**Figure 1:**
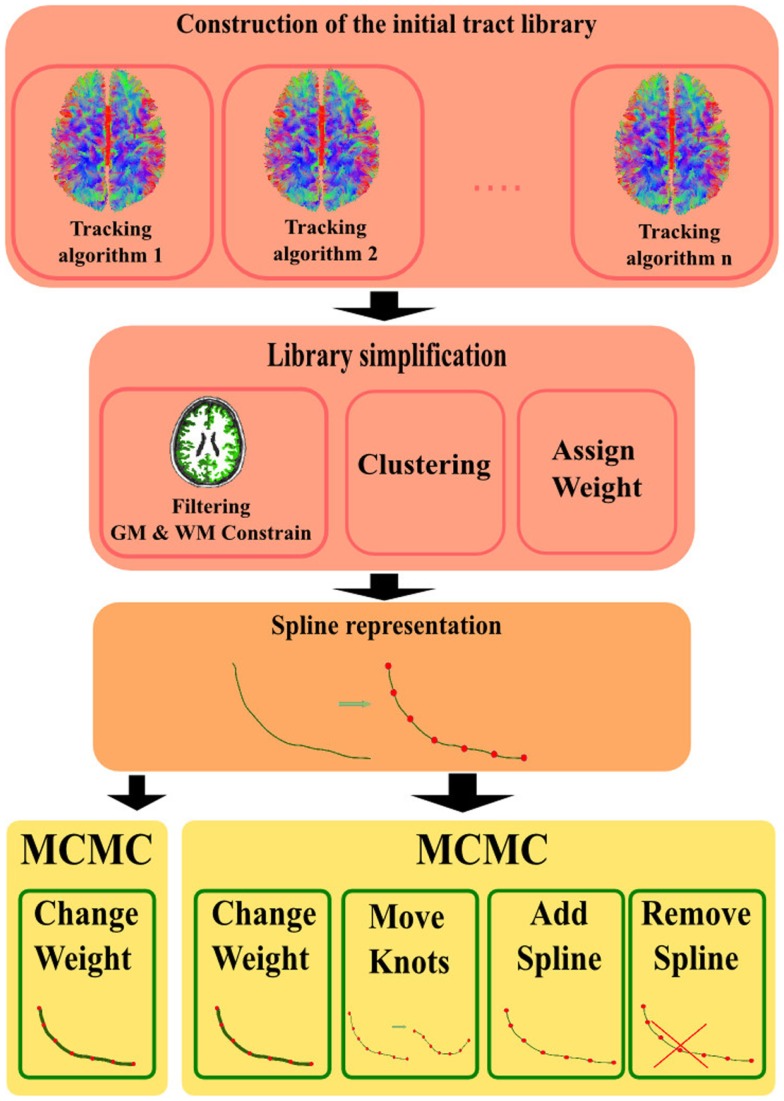
**Flowchart of the different steps in the method: first, construction of the initial tract library by using existing tractography algorithms i.e., (2, 7, 9)**. Second, simplify the library by constraining to anatomical priors, cluster (23), and assign all fibers an initial weight. Third, convert to the model computed to represent the fiber trajectories. Lastly, the MCMC-based method used to optimize the fit of the model vs. the measured data.

The complete process of this method is described in Figure 1 where the different steps of the flowchart are:

1. Construct the *initial library of fiber tracts* by using any classical tractography algorithm (or a combination of them).
2. *Library simplification*: extend the fibers that have stopped just before reaching GM area. Filter out the ones that stopped prematurely in the WM (too far from the GM to be extended), as they do not represent anatomically valid connections (VC). Remove duplicates by using a clustering algorithm and assign an initial weight to every fiber.
3. Represent fibers by means of a parametric representation e.g., a spline to *explicitly enforce anatomical priors* (smoothness, endpoints in GM, etc.).
4. Find the configuration, i.e., tract positions and effective weights, which best explains the measured dMRI data, via *MCMC-based optimization*.

In the following sections, we will provide details concerning each step of our proposed approach.

#### 2.1.1 Construction of the initial tract library

The initial tract library can be estimated by using any tractography algorithm or a combination of them to inherit the pros of each tracking approach. Many of the reconstructed tractograms would have endpoints that do not connect to the GM, as a consequence this will lead to unnecessary computational time for the optimization. The tractography methods used to construct the candidate sets are: the classical line-propagation method based on (2), a front-evolution algorithm similar to (7) and a global approach (9, 10). From now on the three methods will be referred to as, STREAMLINE, SP (Shortest Path), and GIBBS.

For the propagation of the tracts we employed the FOD as local diffusion model, which were provided to us by the Tractometer team (tractometer.org) to ensure the best quality of the local reconstruction. The parameters for tracking were chosen in a way to ensure all true connections in the FiberCup data set. To run the STREAMLINE method we used MRtrix toolbox^1^ with the following parameters: nine seeds per voxel, curvature = 1.0 and a step size of 0.6. Concerning SP an in-house implementation was used to track from every voxel of the boundary GM/WM to another GM region and the information used for the propagation is the same FOD that was used for the STREAMLINE. In the case of GIBBS^2^ the default parameters were used.

#### 2.1.2 Filtering and simplification

To ensure a fast computational time and respect the constraints of having fiber endpoints reaching the GM area, some pre-processing was necessary.

To start with, a clustering algorithm (23) was applied to extract the most representative fibers for each connection, resulting in all the duplicated fibers being removed. The parameters used for the clustering were: a threshold of 3 with a polyline consisting of 25 points. These parameters were chosen to achieve a trade-off between removing as many fibers as possible, but conserving the most significant ones. More details about these parameters can be found in Ref. (23).

Furthermore, since no stopping criteria of the tracking were included in the STREAMLINE algorithm, many fibers stopped before reaching the GM. Due to this, an extension of the fiber endpoints was necessary. However, fiber endpoints stopping more then a voxel before reaching GM would be excluded. A fundamental necessity to run our method is for both endpoints of every initial fiber to lie within a GM region. Lastly, an initial weight was assigned to all the tracts of the tractogram (second block in Figure 1). Further details on how this weight is set is described in the experiment of Section (3.1).

#### 2.1.3 Spline representation to enforce anatomical priors

The aim of this step is to represent the 3D paths between all pairs of regions with a model that grants us the possibility to modify the trajectories, while at the same time preserve the smoothness of the tracts. The model used therefore is a cubic spline namely, Catmull–Rom splines (24). These splines are piecewise cubic polynomial curves that pass through all the control points. The control points were extracted by reducing the number of points describing the polyline and at the same time conserving the shape of the initial curve. To obtain these control points, the Douglas–Peucker algorithm (25) was run on these polylines until seven control points were obtained per fiber. Previous work (26) used five control points to represent the spline, with visual inspection of some tracts in the *in vivo* dataset we decided finally to use seven control points. To stay consistent, we used the same number of control points for the *in vivo* dataset and the FiberCup dataset. However, due to the simplicity of the FiberCup dataset much less control points could have been used. Future improvements of this method would be to adjust the number of control points of the spline depending on its complexity. The main advantages with using this type of splines are firstly that they are cubic, thus easy to compute and fast due to the low number of parameters and secondly that they pass through all their control points. The fact that the entire spline passes through its control points makes it easy to handle. This grants us the possibility to explicitly enforce our anatomical prior information that all fiber endpoints should lie within the GM and all the remaining points in the WM. In case a control point is being outside the GM or WM, we would be sure that the underlying spline would be as well and can therefore exclude it from the sampling scheme. Another advantage of using the splines is that it can include a large variety of paths when the number of control points is large enough and their smooth nature makes them anatomically plausible.

#### 2.1.4 Finding the optimal fiber configuration with MCMC optimization

The global cost to minimize is based on the L2-norm of the distance between the measured signal *D* and the predicted signal with our fiber model, *M*. The goal is to find the most likely model, *M*, given the observed data, *D*, by exploring the distribution of possible solutions from the posterior probability *P* (*M*|*D*). The Bayesian modeling allows the computation of the posterior probability:

(1) $$P\left(M|D\right)=P\left(D|M\right)P\left(M\right)$$

The measured signal is defined as *D*(*x, n*) = *S*(*x, n*)/S_0_(*x*), where *S*(*x, n*) is the diffusion weighted signal at position *x* with gradient direction *n* at a fixed *b*-value. S_0_(*x*) is the non-diffusion weighted signal at a *b*-value of 0. The fiber model, *M*, is composed of a set of segments of equal size. The segment size will depend on the voxel size and a rule of thumb is to set the size of the segment smaller then the voxel size. In our case, the segment size is set to 2 mm in both experiments (FiberCup and *In vivo*) for consistency. As in Ref. (9), the predicted signal is thereafter derived from the segments of the underlying spline (our fiber model) and expressed as following:

(2) $$\rho_{\mathrm{seg}}\left(x,\;n\right)=e^{\left(-{\mathrm{bn}}_{\mathrm{seg}}^{T}D_{\mathrm{wm}}n_{\mathrm{seg}}\right)}e^{-|x-x_{\mathrm{seg}}{|}^{2}∕\sigma^{2}}$$

where, the first exponential is the expression of the tensor according to the the multi-tensor model (27) with the eigenvalues of *D*_wm_ being [2.1; 0.85; 0.85] square micrometers per millisecond for the FiberCup dataset and [1.7; 0.3; 0.3] square micrometers per millisecond for the *in vivo* dataset. The second exponential is the Gaussian distribution centered in *x* of that voxel, where *n*_seg_ and *x*_seg_ are the orientation and position of the segment, respectively. After some experimental testing, a *σ* = 0.5 was chosen and used throughout all the experiments.

Every fiber has assigned to it a unique weight, denoted *w*; importantly, the weight assigned to a particular fiber is used to scale the contribution of all segments along that particular fiber, i.e., the weight of the fiber does not vary along its length.

The total signal contribution of a fiber in a voxel is basically the sum of all its segments contribution multiplied by the weight *w* ≥ 0.

(3) $$\rho_{M}\left(x,n\right)=w\underset{seg}{\sum }\;\rho_{\mathrm{seg}}\left(x,\;n\right)$$

The weights of all fibers, together with the control points are the unknown parameters that are to be optimized using our method. The property of these two parameters are very helpful in reducing the search space since: (a) thanks to the weights, less fibers can be used to represent a connection between a pair of region (b) an entire fiber (spline) can be described using a few number of control points.

Finally, the energy function to minimize is:

(4) $$E\left(M,\;D\right)={∥\rho_{M}-D∥}_{L_{2}}^{2}$$

This expression is now optimized using a Metropolis-Hastings sampler with a simulated annealing (SA) (28) approach. As long as *E* (*M*′, *D*) is smaller than *E* (*M, D*) the new state is accepted, otherwise it is accepted with regards to this criteria:

(5) $$e^{-E\left(M,D\right)∕T}>R\left(0,\;1\right),$$

where *T* is the system temperature and *R* (0, 1) is a random number in the interval [0, 1]. The SA involves a decrease in the system temperature *T* from a high starting temperature to a low final temperature. If *T* is large, bad transition states are accepted and a large part of the solution space is accessed. This will allow the system to explore more of the solution space in the beginning of the process and successively restrict it until it reaches the final temperature.

The proposals consist of:

- Changing weight of a fiber.
- Moving, either by moving the control points or translating the whole fiber.
- Adding a fiber.
- Removing a fiber.

The process works as follows: start with the current model state *M*, modify the state using one of the four proposals. The modification of the state is carried on by sampling the position of a control point from a normal distribution of GM or WM volume. The weights are as well sampled from a normal distribution in the range of 0 and 1. The new state is now denoted *M*′.

The probabilities of picking a certain proposal are fixed after a number of experiments according to the following: changing weight of a fiber = 40%, move a fiber = 40%, add a fiber = 15%, and remove a fiber = 5%.

### 2.2 Data and experiments

#### 2.2.1 Phantom

The first experiment was performed using data acquired from a physical diffusion phantom, which was designed and built for the FiberCup Contest in MICCAI 2011 (15). We used the dataset with a *b*-value of 1500 s/mm^2^, 64 diffusion directions and an in-plane resolution of 3 mm × 3 mm with a slice thickness of 3 mm. For further details about this data set, we refer to footnote 3^3^.

Before starting our optimization method, we first search for the optimal weight for each initial tractogram (STREAMLINE, SP, and GIBBS). By computing the cost using Eq. 4 we retrieved the optimal weight considering the lowest cost (second block in Figure 1). Next, we ran our MCMC method, yellow blocks in Figure 1, by using first only one of the four proposals namely, the change of weight (left yellow block in Figure 1). The aim for this test was to see how big the impact the weight change had on fitting the model to the data. Thereafter, we ran our method including all the four proposals as described in Section (2.1.4) and shown in Figure 1, last and far right block.

To evaluate how well the final obtained model fit the measured data, we used the normalized mean-squared error (NMSE) defined as:

(6) $$\mathrm{NMSE}=\frac{{∥\rho_{M}-D∥}_{2}^{2}}{{∥D∥}_{2}^{2}}$$

Furthermore, we carried out the analyses by attempting to see if we managed to classify the computed “connection strength” as valid and invalid pathways. The “connection strength” is defined here as the sum of weights of all fibers within the bundles connecting a pair of regions in the GM. Usually, a bundle in traditional fiber tracking algorithm would have fibers with the same weight (one), whereas in our case every fiber has an unique weight assigned to it. The classification algorithm used is the *k*-mean classification algorithm given two classes, one class representing the connections with a very low “connection strength” and the other representing the strong connection. This experiment can however only be conducted on the FiberCup dataset since the ground-truth (GT) is known.

Thereafter, a quantitative comparison was performed using the Tractometer methodology proposed in Ref. (14). It is an evaluation system for tractography pipelines with a particular emphasis on global connectivity. The global connectivity scores used here are:

- Valid Connections, connections that connect a pair of ROIs that are known to be true with respect to the GT.
- Invalid Connections (IC), connections that do connect a pair of ROIs that are known to NOT to be true with respect to the GT.
- No Connection (NC), connections that do not connect any pair of ROIs or only one endpoint does.
- Valid Bundles (VB), bundles connecting a pair of ROIs that are known to be correct considering the GT.
- Invalid Bundles (IB), bundles connecting a pair of region that are known to be incorrect.

Valid connections, IC, and NC are reported in percentage and are per-fiber contribution, whereas VB and IB are reported as absolute values (e.g., only a single reconstructed trajectory between two ROIs can be determined to be VB or IB).

Lastly, the coefficient of variation (CV) of the “connection strength” was computed over 10 runs for every tractography algorithm to reveal the reproducibility of the methods.

#### 2.2.2 *In vivo* human data

In addition to the experiments on the FiberCup data, we tested our method on an *in vivo* human brain. This data were acquired on a healthy subject with the following acquisition parameters: a *b*-value of 2000 s/mm^2^ and 150 diffusion direction and a voxel resolution of 2 mm × 2 mm × 2 mm. The GM and WM mask were obtained using Freesurfer from a T1-weighted image. However, any segmentation of the GM and WM could be used. Furthermore, since our method is connectivity oriented and since Freesurfer provides us with GM parcellated into regions of interest, we incorporate this prior information already from start.

As for the FiberCup data, we first searched for the initial weight. Furthermore, the method is tested by only optimizing the weight of the fibers and thereafter by using all the suggested proposals as described in the method section.

## 3 Results and Discussion

### 3.1 Initial weight optimization

**Figure 2:**
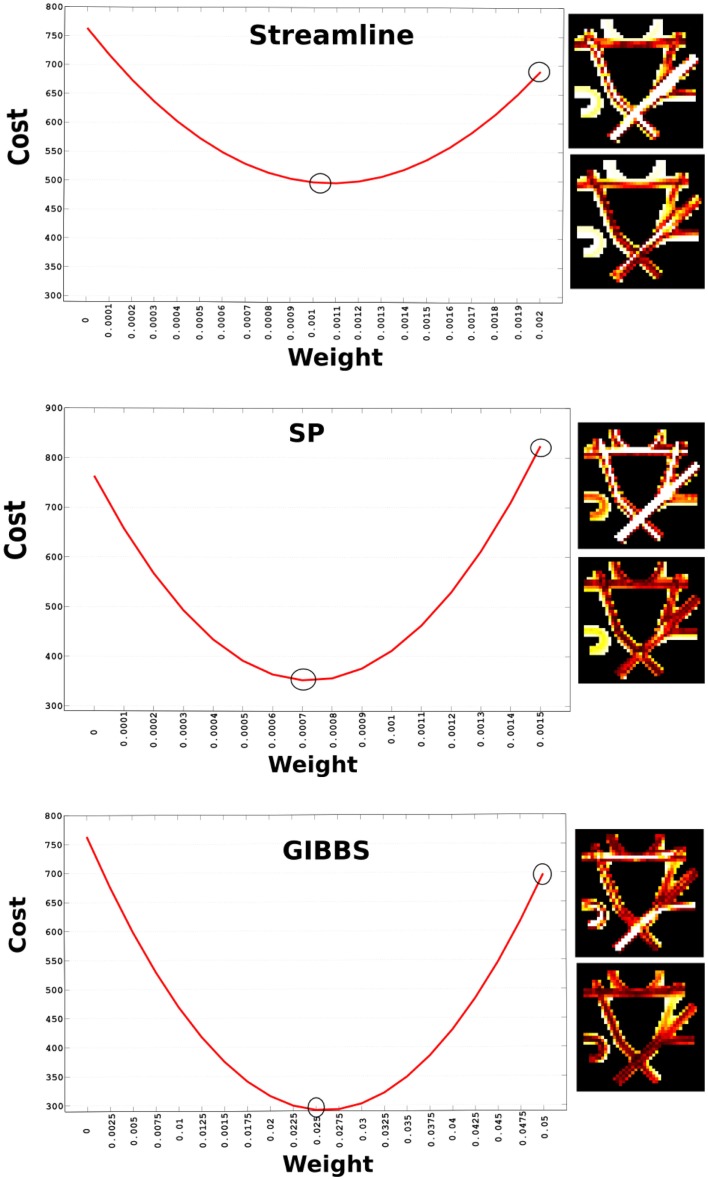
**Cost vs. initial weight for the three different candidate fiber sets**. The two circles indicate the weight used to generate the NMSE maps displayed to the right of the plot. The upper circle corresponds to the upper NMSE and the lower one to the lower NMSE map.

Every tracking algorithm might produce different densities and reconstructions of fibers, which do not only depend on the algorithm itself but as well on the parameters used. Therefore, an appropriate global scaling needs to be found to enable the comparison of the initial fiber set. Since these tractography methods do not provide a weight for every fiber, we need to search for it. Figure 2 shows how the cost changes depending on the weight. We found out that the optimal weight for the STREAMLINE was 0.0011, for the SP 0.0007, and GIBBS 0.025. These weights were obtained by computing the global cost as expressed in Eq. 4 alternating weights between 0 and 1. The weight generating the lowest cost would then be used as the initial starting weight for that tractogram. Future improvements would be to solve this part with a convex formulation as in Ref. (22), to ensure a unique starting weight for every single fiber.

Furthermore, a map of NMSE shows how well we could already fit the signal just by adapting weight to every tractogram. These numbers make sense considering the number of fibers every method generates, for example GIBBS, which only generated about 250 fibers needs a higher initial weight compared to the other candidate sets. However, it is important to keep in mind that the value of the weights will not only depend on the number of fibers but as well on how they are distributed across the WM. In addition, multiple fibers following exactly the same path can be compactly represented by only one trajectory and its corresponding weight will be the sum of all of them.

### 3.2 MCMC proposal evaluation

**Figure 3:**
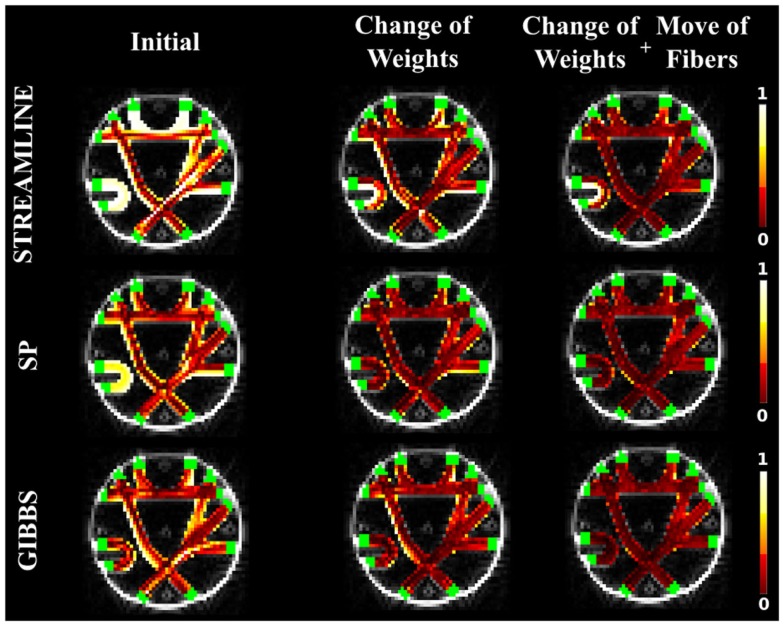
**The NMSE maps overlayed onto the b0 diffusion images and the regions of interest in green**. The columns represent the initial solution (left), the final solution when using only the weight change proposal (center), and the final solution when all the four proposals were used (right). The rows correspond to the different initial candidate sets: STREAMLINE (top), SP (middle), and GIBBS (bottom).

Figure 3 shows NMSE maps of the FiberCup data comparing the error of the initial candidate set to the error after our method is applied. Each row corresponds to different initial candidate set used. The columns represent from left to right, the NMSE in every voxel for the initial starting set (left), the results of using our method but only using the weight change proposal and finally (right), the results by using all proposals (bottom far right block in Figure 1). Table 1 compares the cost from each initial set to the cost after running our method using only the weight change proposal and all four proposals. In all cases, we see that our method reduces the cost from the initial setup and that using all proposals gives the best results. These results clearly show the importance of not only changing the fiber weights, but also to give the fibers the flexibility to move. Another interesting observation is that using the fiber movement proposal, we are able to distribute fibers in the border areas on the FiberCup data, which have proven to be challenging due to the so called stopping criteria in fiber tracking (13). Having the priors of fibers starting and ending in the GM is very important in this aspect, since we will be optimizing a set of connectors that are anatomically correct and disregard all small portions of fibers ending in the WM. These small portions of fibers might describe the signal better locally (9), however, if it does not reach the GM then it cannot be considered a fiber anymore.

**Table 1:** **Number of fibers in the initial set of the FiberCup data, initial starting weight assigned to every fiber and the initial cost**.

	Number of fibers	Initial weight	Initial cost	Change of weights	Change of weights + move of fibers
STREAMLINE	2463	0.0011	496	304	186
SP	6870	0.0007	352	194	148
GIBBS	250	0.025	292	204	134

*The two last columns show the final cost when first optimizing the weight only and when using all four proposals*.

### 3.3 Classification of fiber bundles

The previous section showed that we can greatly reduce the error between the measured data and different initial candidate sets of fibers using our method, this section evaluates our methods ability to correctly classify bundles.

**Figure 4:**
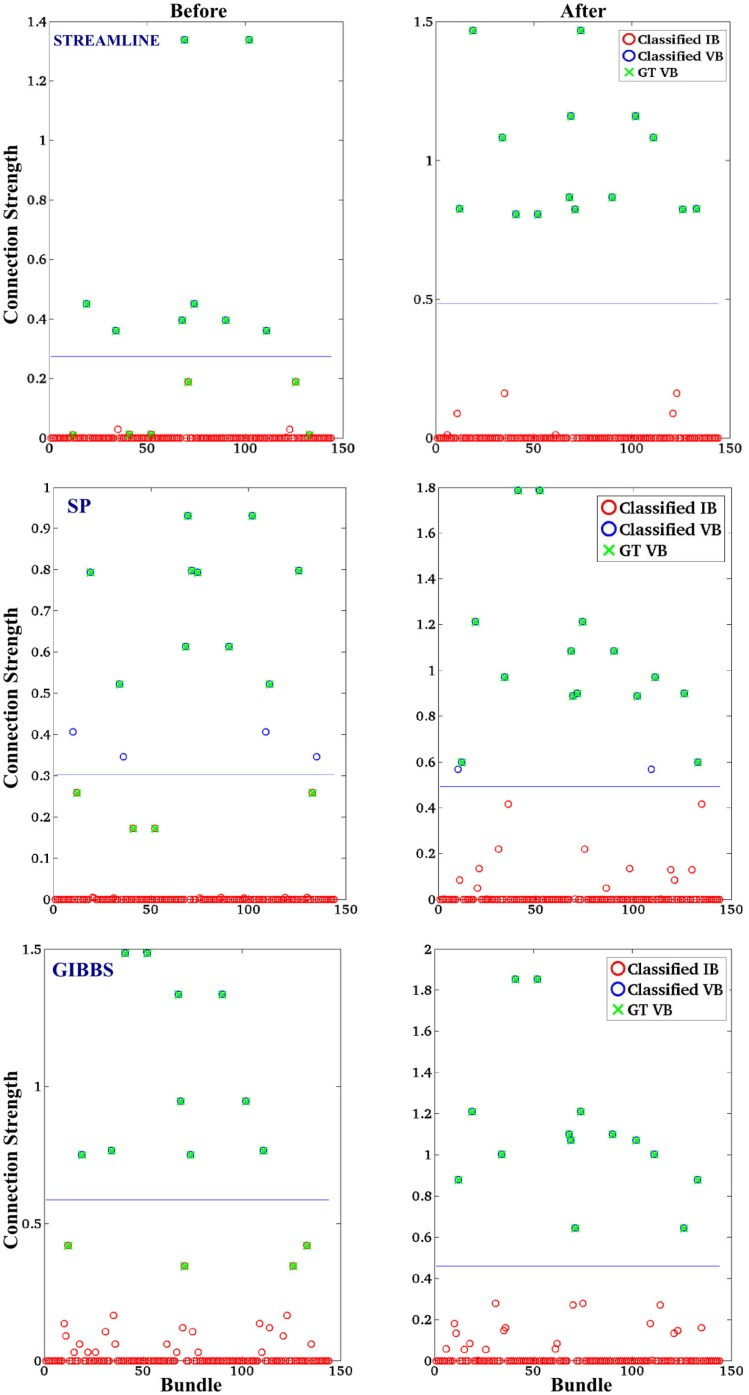
**The classification of valid bundles (VB) and invalid bundles (IB)**. The initial candidate derived with: STREAMLINE, SP, and GIBBS. The first column indicated the classification on the tractography method before our approach and the second column shows the classification after. The connection strength is the median over 10 runs for each method and *x*-axis represents the bundles connecting every ROI (12*12) in the FiberCup phantom.

In order to obtain the most representative “connection strength” (defined in 2.2.1) of every bundle of all the methods, we extract the median value over 10 runs of the “connection strength” for each bundle. Figure 4 shows the classification results of using a *k*-mean classification algorithm of all the bundles in order to separate VB (high “connection strength”) and IB (low “connection strength”), as defined in the next Section (3.4). The final results that were based on the STREAMLINE or GIBBS as an initial set, were classified perfectly. However, in the case of SP as an initial solution, one of the bundles was incorrectly classified. This specific bundle has previously shown to be difficult to classify correctly (29).

### 3.4 Fiber bundles and tractometer scores

To derive some further quantitative evaluations, Table 2 demonstrates the results of the Tractometer Scores (14). The computational time required to obtain these results is about 8 min, including the polyline to spline conversion and the MCMC optimization procedure. We based these results on an average series of 10 runs of our method using the STREAMLINE as initial set and compare our results to GIBBS. With our method we managed to recover the whole 100% of VC, compared to GIBBS 21.92% and in which as well lead to the results of IC that is 0% in our case and 3.48% for GIBBS. Furthermore, it is worth noting that GIBBS generates 74.6% fibers that do not connect to two regions of interest (NC). While in our case we disregard such fibers since they are not anatomically plausible. In addition, the results from the initial candidate set shown here, are based on tractograms where the endpoints of the fibers have already been extended to reach the GM. In the case of STREAMLINE, only one VB was found before the extension for instance. Furthermore, it is important to note that the results from our method are based on the outcome of the classification performed in the earlier section. Whereas, the initial tractograms (extended and filtered) are being evaluated in their original form since the classification fails in their case.

**Table 2:** **Quantitative. comparison of our approach run on the different initial tractograms using the scores proposed in Ref. (14)**.

	VC (%)	IC (%)	NC (%)	VB	IB
STREAMLINE extended	23.70	1.68	74.62	7	2
STREAMLINE filtered	98.96	1.04	0	7	2
STREAMLINE + our approach	**100**	**0**	**0**	**7**	**0**
SP filtered	84.18	15.82	0	7	6
SP + our approach	**93**	**7**	**0**	**7**	**1**
GIBBS extended	21.92	3.48	74.60	7	12
GIBBS filtered	86.68	13.32	0	7	12
GIBBS + our approach	**100**	**0**	**0**	**7**	**0**

*The results are reported as an average over 10 runs. Extended, the extension of the endpoints to reach the GM and filtered, considering only fibers connecting both endpoints to GM. The bold highlights the results of our approach*.

**Figure 5:**
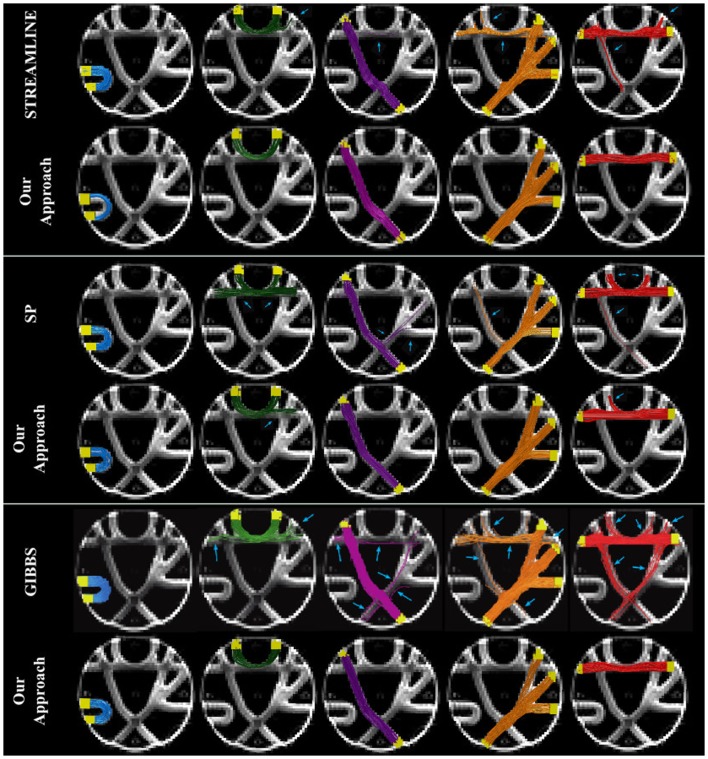
**Qualitative evaluation of fiber bundles in the FiberCup data**.

Figure 5 shows the seven golden standard bundles of the FiberCup phantom, where the 4th column correspond to the branching structure that is collapsed into a single image. We compared our results to the initial tractogram that it was based on and have highlighted with a blue arrow the invalid or not connecting fibers (IC and NC). Fibers that are shown here have at least one endpoint connected to the GM region (yellow). We can already observe how our method suppressed all the invalid and no connecting fibers that are known to be incorrect according to the GT.

Concerning the reproducibility, the CV was computed for every cell in the connectivity matrix (“connection strength”) over 10 runs for every method. Table 3 demonstrates the average of this CV. We can clearly see how the reproducibility is improved with our method. The 10 runs of streamline tracking based on exactly same the parameters and the very same local reconstruction (fODF) still generates a CV of 2.7% while if we run our method using the same STREAMLINE tractogram as an initial starting set we end up with a CV of 0.3%.

**Table 3:** **Coefficient of variation (CV) of the connection estimates as quantified by the three tractography methods**.

	STREAMLINE	SP	GIBBS
Before	0.027	0*	0.28
After	**0.003**	**0.0045**	**0.01**

Results represented both before and after using our approach. *Denotes that CV for SP is 0 since it is a deterministic approach. The CV of our method is highlighted in bold.

Since the same fODF is used and the SP is a deterministic approach, the CV is 0 and indicated with *. GIBBS seems to be the method that was least reproducible (CV = 28%), but applying our method to it we end up with a CV of 1%.

### 3.5 *In* *vivo*

**Figure 6:**
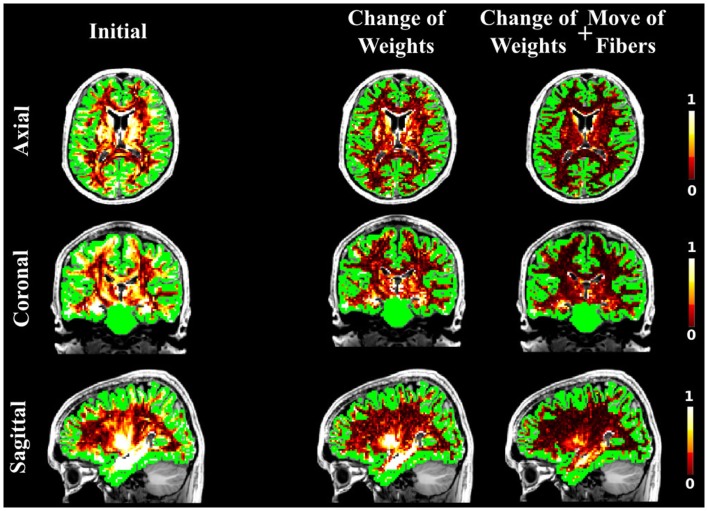
**The NMSE maps between the acquired *in vivo* dMRI image and the signal generated with the initial set derived from the STREAMLINE tracking**. The maps are overlayed onto the T1-weighted and GM segmentation in green. The first column shows the NMSE for the starting set, the second column shows the results when running our method by only changing weights, and the last when all our proposals are used in the optimization.

**Figure 7:**
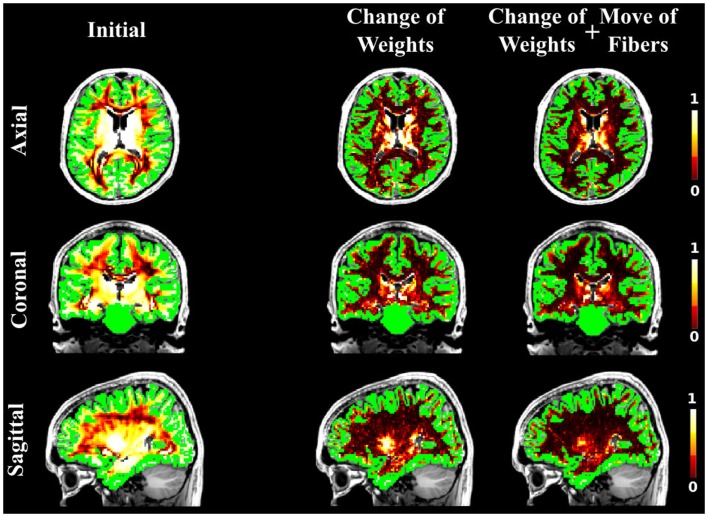
**The NMSE maps when SP was used as initial candidate set**. The maps are overlayed onto the T1-weighted and the GM regions. The first, second, and third columns illustrate the NMSE of the initial set, changing the weights only and finally using all our four proposals.

**Figure 8:**
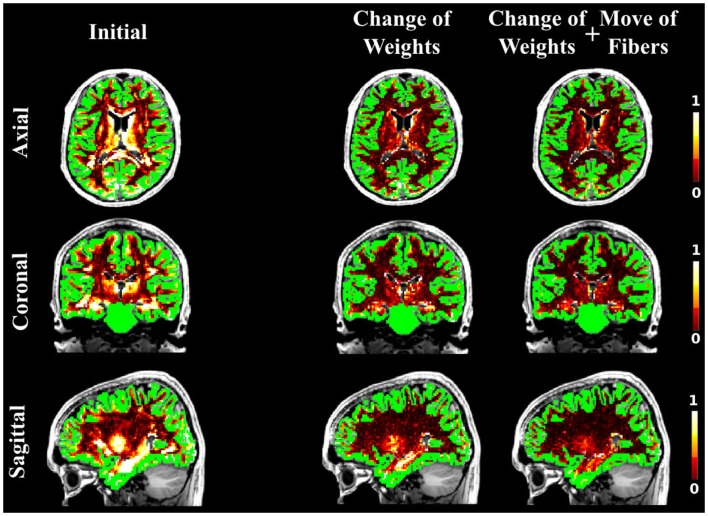
**The NMSE maps overlayed onto the T1-weighted and the GM regions**. The initial set was estimated using the GIBBS tracking algorithm, which was then used as an initial set for our approach. The results when optimizing the weights only (second column) and when all suggested proposals are used (third column) are compared to the initial set (first column).

As in the case of the FiberCup data, we computed the NMSE for the *in vivo* data as well. Figures 6, 7, and 8 show the NMSE maps between the modeled and measured signal overlayed onto the T1-weighted and the GM regions (green) for the three different views of the brain (Axial, Coronal, and Sagittal). The first column illustrates the NMSE of the initial candidate set, when the same initial weight was set to every fiber of the tractogram. To compute these initial tractograms it took about 20 min for the STREAMLINE, 1 h for the SP, and 12 h for the GIBBS. We can clearly see that NMSE decreases when our approach is applied: first, changing only the weights as shown in the second column of the figures and, even more, when all our four proposals are used, as demonstrated in the far right column of Figures 6, 7, and 8. Both Figures 6 and 7 clearly illustrate the need of moving the trajectories of the tracts to better explain the measured diffusion MR signal. Figure 8 on the other hand shows already promising results with a visual inspection of the maps when only the weights are optimized. However, it is worth noting that the final cost still improves considerably when the fibers are being moved as well. It is as well worth to recall the computational time it takes to obtain one full tractogram using GIBBS, 12 h, considering our method that took about 25 min on this dataset no matter what the initial tractogram was. However, the computational time will always depend on the parameters used, decreasing for instance the *σ* for the Gaussian smoothing would speed up the process extensively, but the price to pay is the restricted search space.

**Figure 9:**
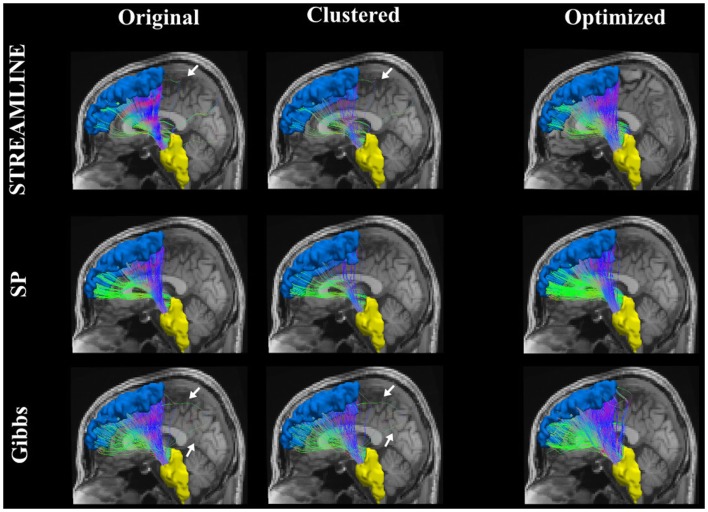
**The pathway connecting the brainstem to the superior frontal**. The far right column shows the original tracts, the center column is the result after simplifying the data set using QuickBundles (23), and the last column shows the results from our optimization procedure.

**Figure 10:**
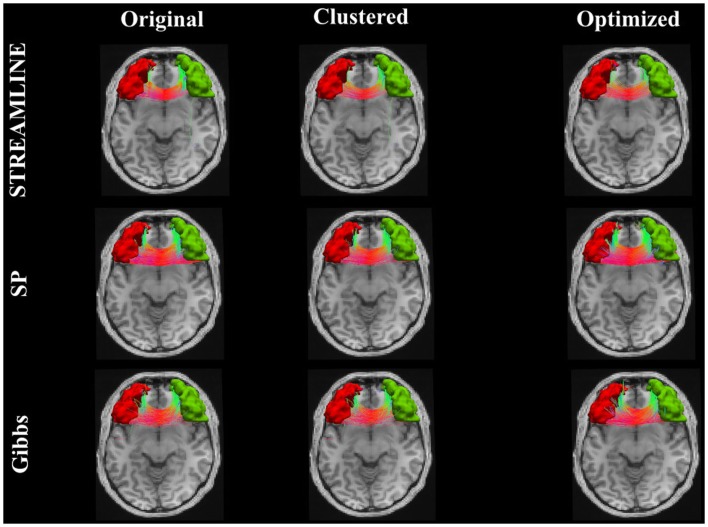
**The pathway connecting the right and left rostral middle frontal regions**. The first column illustrates the initial tracts, the second column shows the results when the clustering algorithm (23) was applied. The last column shows the final results with our approach.

Figure 9 shows the connecting tract between the brainstem and the left superior frontal. This figure illustrates and compares the tracts from the original dataset where the endpoints were extended to reach the GM regions (here, the brainstem and the superior frontal). The second column demonstrates the dataset that was used as our initial set after applying the clustering algorithm and lastly the third column shows the results when our method is applied. Further on, Figure 10 shows an inter-hemispheric connection between the right and left rostral middle frontal regions where the color coding of the tracts indicates the fiber direction. We can clearly see that even though we start out with a quite sparse tractogram (clustered) we manage to reconstruct fiber tracts that appear to be located according to the underlying anatomy. We can as well observe in Figures 9 and 10 how our final reconstructed pathways have no big dependency on the initial tractogram. In addition, each and every one of our fibers has a weight assigned to it, although it is unfortunately not displayed.

Despite the fact that our method manages to fit the data well when all our proposals are used, it is still based, as most existing approaches [e.g., Ref. (9)], on a simple model that does not consider all existing diffusion compartments that contribute to the signal in each voxel. Therefore, a possible future extension of this method is to develop an alternating scheme, which combines this work with our previous approach (22), with the aim to inherit the benefits of both approaches. On one hand, we might be able to estimate very efficiently the contribution of each tract to the image by exploiting the convex formulation in Ref. (22); on the other hand, using the algorithm proposed in this work, we could now add the possibility to adapt the position of the tracts, thus being less sensitive to the initial set of candidate tracts (22).

## 4 Conclusion

We have presented a method, which incorporates anatomical priors by using the spline model to describe the 3D pathway of the brain and furthermore a quantitative measure of the fibers is introduced. We have demonstrated our optimization method on both synthetic data (FiberCup) with a known GT that allowed us to verify the accuracy of the method and as well on *in vivo* data. Our findings clearly state the importance of including the anatomical priors and the quantitative measurement (weight) of the fibers into our optimization method. We believe that that this new approach presented here, will be of big value for the community performing connectivity analysis on the human brain data.

## Conflict of Interest Statement

The authors declare that the research was conducted in the absence of any commercial or financial relationships that could be construed as a potential conflict of interest.

## Footnotes

^1^http://www.brain.org.au/software/mrtrix/

^2^http://www.uniklinik-freiburg.de/mr-en/research-groups/diffperf/fibertools.html

^3^http://www.lnao.fr/spip.php?rubrique79
